# Tuberculosis

**Published:** 1894-03

**Authors:** Williamson Bryden

**Affiliations:** Boston


### TUBERCULOSIS.

*Mr. Editor :*

In view of the recent action of the Massachusetts Veterinary Medical Society endorsing the placing of tuberculosis under the Contagious Diseases of Animals Act, I wish to record my opposition.

I do not believe that tuberculosis is either hereditary, contagious or infectious, or that it ever could be extended in any such a way. There are more *degrees* of it than there are tissues to become affected. Up to a certain point it is quite curable. Only in a very few cases of neglected animals is slaughter necessary. In the cow it is entirely caused by over-milking and over-breeding ; she is bred too early and often, and the animal's system is drained and impoverished until she cannot rally and restore herself as she would if her milk-giving was regulated to 4, 6 or 8 months—until she matured, and her breeding was carefully managed.

Her crosses, instead of being with heavy milkers, must be with well-bred, semi-wild range or prairie cattle, managed with care and judgment. The heavy milker crossed with the great milker is a great mistake. The hen that is forced to lay eggs for the market and for the incubator will also become similarly affected, and so will the monkey, and so will the fruit tree.

What will be thought of the cattle commissioners of a State like Massachusetts, that allows her cows to be quarantined by Maine and New Hampshire, without immediately ordering Maine and New Hampshire cattle similarly restricted and kept from entering Massachusetts. I would not allow an animal killed, as I believe that only a very few are unfit for food.

Why it exceeds in heartlessness the terrible slaughter of innocents for contagious pleuro-pneumonia, when at least $\frac{3}{4}$ of those destroyed never had the disease and the veterinary profession refused to investigate it after Professor Williams had told them of their mistake.

It can be transmitted just as spavin, ring-bone, navicular disease. It can be reproduced in animals that never had a taint of it.

BOSTON. WILLIAMSON BRYDEN, V.S.

